# Total Environmental Impact of Three Main Dietary Patterns in Relation to the Content of Animal and Plant Food

**DOI:** 10.3390/foods3030443

**Published:** 2014-07-25

**Authors:** Luciana Baroni, Marina Berati, Maurizio Candilera, Massimo Tettamanti

**Affiliations:** 1 Primary Care Unit, District No. 4, U.L.S.S. No. 9, Treviso 31100, Italy; E-Mail: lbaroni@ulss.tv.it; 2 Nutrition Ecology International Center (NEIC), Torino 10100, Italy; E-Mail: marina.berati@mclink.it; 3 Department of Mathematics, University of Padua, 35121 Padova, Italy; E-Mail: candiler@math.unipd.it

**Keywords:** Life Cycle Assessment (LCA), Dietary Guidelines, environmental impact

## Abstract

Based on a review of the most recent available scientific evidence, the new Dietary Guidelines for Americans 2010 (USDA DG) provide information and advice for choosing a healthy diet. To compare the environmental impacts of, respectively, omnivorous (OMN), lacto-ovo-vegetarian (LOV) and vegan (VEG) dietary patterns as suggested in the USDA DG, we analyzed the three patterns by Life Cycle Assessment (LCA) methodology. The presence of animal food in the diet was the main determinant of environmental impact. The major impact always stemmed from land and water use. The second largest impact came from energy use. Emission of toxic inorganic compounds into the atmosphere was the third cause of impact. Climate change and acidification/eutrophication represented other substantial impacts.

## 1. Introduction

The evidence for a link between high consumption of meat and other animal foods and poor health outcomes is growing. The main contributing factors are likely to be the high saturated fat content in animal products, the high salt content in processed foods, and the fact that the consumption of animal products limits the consumption of health-promoting foods such as fruit, vegetables, nuts, beans and grains [1,2,3,4,5,6,7,8,9,10,11,12,13].

According to the World Health Organization (WHO), malnutrition affects one in every three people worldwide; it is present in all age groups and populations, and plays a major role in half of the 10.4 million annual child deaths in the developing world; malnutrition also plays a role in causing disease and disability in the children who survive [14,15].

A European study tackled the problem of sustainable food production and assessed the environmental impact of human food consumption and its related processes [16]. The study assumed as the basis for calculations a complete diet (defined as the total amount of food that one single person eats in one week), whereas previous research had been mainly limited to single foods or to specific comparisons [17,18,19].

Its results confirmed the need to educate people living in developed countries to shift their eating habits towards an increase in the direct consumption of plant foods.

The 2010 Dietary Guidelines for Americans, USDA DG [20], released in 2011, provide information and advice for choosing a healthy diet; namely, one that focuses on nutrient-dense foods, and that contributes to achieving and maintaining a healthy weight. They are meant to be used in developing educational materials for the general public, and to aid policymakers in designing and carrying out nutrition-related programs, including federal nutrition assistance and educational programs. To get the full benefit, it is recommended that individuals abide by the USDA DG recommendations in their entirety, as part of an overall approach to a healthy lifestyle.

Although the USDA DG 2010 declare that the recommendations are based on a review of the most recent available scientific evidence, it must be noticed that there is not unanimous agreement among the nutrition science community that the recommendations do in fact reflect the *best* available scientific evidence. In particular, Harvard School of Public Health judges that the USDA recommendations do not reflect “the best eating choices” and proposes its Healthy Eating Plate, an eating guide for omnivorous people that is more selective on the quality of diet and more limited on the amount of dairy food [21].

The aim of the present study is to further explore and compare the environmental impacts of different but “homogeneous” food patterns. We chose to use the omnivorous USDA food patterns (OMN) and their vegetarian adaptations, respectively, lacto-ovo-vegetarian (LOV) and vegan (VEG) patterns, as suggested in Appendices 7, 8 and 9 of the USDA DG [20].

The “omnivorous” (OMN) food pattern includes animal flesh and animal products, and any plant food. The “lacto-ovo-vegetarian” (LOV) food pattern includes any plant food and milk, dairy products and eggs, while excluding any type of animal flesh (meat, poultry or fish); the “vegan” (VEG) food pattern is a plant-only diet which excludes any food of animal origin: not only meat, poultry and fish but also milk, dairy and eggs. The three dietary patterns suggested in the USDA DG are recommended as healthy and nutritionally adequate [20].

The assessment of the environmental impact of the dietary patterns was conducted using the Life Cycle Assessment (LCA) method, an internationally standardized procedure (ISO 14040) [22].

## 2. Materials and Methods

### 2.1. Life Cycle Assessment

The analysis was performed using the Life Cycle Assessment (LCA), an objective procedure for the evaluation of the energy and environmental impacts of a process or activity. More relevant results stem from the application of the LCA methodology to the total environmental impact of a complete dietary pattern, rather than from its partial application to single steps or single impact subcategories of a production process, or to specific food items. The LCA approach allows to:

1. systematically estimate the complete environmental consequences and analyze all the energetic and material exchanges occurring in the environment, and
2. quantify the various emissions into air, water and land in every life cycle phase, and
3. detect any significant change in the environmental effects in an objective way, and
4. estimate the effects of material consumptions and environmental emissions on the health of human beings and on the ecosystem as related to food production.

Usually, it is carried out through the identification of the energy and raw material consumption and the release of waste into the environment: the assessment includes the whole life cycle of a real process or a real activity, from the extraction and processing of raw materials to the production, transportation, distribution, use, reuse and recycling, and final disposal.

Since the aim of our study was to evaluate the pure food-related impacts, focusing on theoretical diets and keeping other variables fixed (*i.e.*, not to compare locally-produced, low-impact foods *versus* imported, high-impact foods), we did not consider any difference related to geographical zone or transportation; the import-export food fluxes have not been considered, nor related emissions during cooking and storing in the household/in restaurants have been taken into account. The system boundaries included the following steps in the process chain: agricultural production, processing and packaging.

According to ISO 14040 standards for LCA [22], four phases have been performed: 1, Goal and Scoping; 2, Life Cycle Inventory; 3, Life Cycle Impact Assessment, LCIA; 4, Life Cycle Interpretation.

#### 2.1.1. Goal and Scoping

The goal of the study was to compare the environmental impact of the OMN, LOV and VEG dietary patterns proposed in the USDA DG for Americans [20]. We took into account only food produced by intensive, non-organic farming, both because this is, and is likely to remain, the most widespread method, and because previous research had already shown that the production method (non-organic or organic) [16] and transportation [23] have much less influence on the overall environmental impact compared to the source (animal or plant) of the food. The software we selected to carry out the Inventory Analysis and the Impact Assessment was SimaPro 7.3.3 [24].

#### 2.1.2. Life Cycle Inventory

In this phase, which is the core of any LCA, all data were collected, and a model representing the whole life cycle of the products, processes and activities was prepared. In some cases, as stated in the USDA DG, it was necessary to subsume individual foods into overall categories, in order to compare new results with existing databases or previous literature which, sometimes, presented simplified data for “fruit”, “vegetables” and “cheese”.

Input/output data on processes in the food sector have been collected from a variety of sources. Data on production in agriculture and fishery have been determined by a “top-down” approach, where statistical data on a national level have been broken down to represent specific processes. Specific data collection was performed from textbooks/scientific papers describing specific case-studies [16,18,19,25,26,27,28,29,30,31,32,33,34,35,36,37].

#### 2.1.3. Life Cycle Impact Assessment (LCIA)

In the LCIA phase, the collected data were used to evaluate the various environmental impacts, and to quantify the impact of each single process on the overall damage.

The elements necessary to this assessment are:

1. selection of impact categories (environmental effects) and of the environmental indicators representing them;
2. attribution of the results of inventory analysis to the selected impact categories (classification), according to the effects they exert or may exert on the environment.

The software assigns to each component of the diet a “weight”, *i.e.*, an a-dimensional value, which represents the intensity of the effect that each component exerts on the environment. The “total weight” of each diet, called single score, is expressed in points (*Pt*), the unit of measure used by the software to assign a numeric value to the overall environmental impact of the diet. The higher the “score” in *Pt*, the higher the damage to the environment.

In order to obtain a complete analysis, to facilitate comparison with data from other studies, and to minimize bias, the assessment phase has been conducted using all the indicators made available by the SimaPro software.

##### Ecoindicator99

A damage-oriented indicator that analyses the following impacts, which can be further categorized according to three large damage categories:

1. damages to human health (substances which have a negative impact on respiration, organic and inorganic compounds, carcinogenesis, climate change and ozone, ionizing radiations);
2. damages to ecosystems quality (ecotoxicity, acidification and eutrophication);
3. damages to resources (use of primary resources—land and water—and of fuel).

##### Ecopoint

An indicator designed to evaluate the impacts due to the release of chemicals into the environment (NO*x*, SO*x*, NH_3_, CO_2_, Metals, COD, DUST PM_10_,* etc.*). The Swiss Ecopoints 1997 (environmental scarcity) is an update of the 1990 method, based on actual pollution and critical targets, derived from the Swiss policy for Environment. It comes in 3 versions, with identical evaluation and indicator values but different in the normalization factor; the version number 2 is used in SimaPro [38,39].

##### EDIP

A method adapted for LCA food database projects, representing the most used and widespread indicator to evaluate different forms of toxicity (global warming, acidification, eutrophication, ecotoxicity, human toxicity).

The LCIA was carried out three times, once for each indicator. LCA experts assume a general uncertainty of 10% to 20% in the results [40].

#### 2.1.4. Life Cycle Interpretation

In this phase, all the results of the Inventory and/or of the Impact Assessment were processed, according to the objective and purpose of the research, in order to formulate conclusions and recommendations. It is the final phase of an LCA and its purpose is to propose the necessary changes to reduce environmental impacts.

### 2.2. Diets

Beside omnivorous (OMN) dietary patterns (Appendix 7), USDA DG suggest vegetarian adaptations, respectively lacto-ovo-vegetarian (LOV) and vegan (VEG) patterns (Appendices 8 and 9), from 1000 to 3200 kcal, that meet the nutritional needs of a well-balanced diet for healthy individuals [20].

The three food patterns share a main overlapping component, which is the same for each type of pattern for kind and amounts of suggested foods, and which is composed by “fruits”, “vegetables” and “grains” groups. The three patterns differ in the “oils” group (for amount of servings and the presence of fish oil in the OMN pattern), “dairy” group (which is substituted with non-dairy milk and derivatives for VEG pattern), and “protein foods” group. The latter, in each of three patterns, includes nuts, seeds, and soy products, in different amounts. In the OMN pattern, the “protein foods” group includes also seafood, meat and poultry, while in LOV and VEG patterns it includes again the “beans and peas” subgroup (already present in the “vegetable” group, but in addition to the amounts recommended in it). Moreover, it is important to note that “nuts and seeds” and “soy products” represent a single protein food subgroup in the LOV and VEG patterns, while in the OMN patterns they are aggregated in the “nuts, seeds and soy products” protein subgroup; similarly, “eggs” represent a single protein food subgroup in the LOV patterns, while they are aggregated in the “meat, poultry, eggs” protein food subgroup in the OMN patterns. The relative contribution of each food of these aggregated categories in the OMN patterns has been calculated on the basis of food consumption reported in the FAO database [28].

#### 2.2.1. “*Whole Diet*” Study

To compare the environmental impact of the OMN, LOV and VEG dietary patterns proposed in the USDA DG for Americans, we choose for each pattern three plans with calorie intakes of 1600, 2400 and 3200 kilocalories respectively: 1600 OMN, 2400 OMN, 3200 OMN; 1600 LOV, 2400 LOV, 3200 LOV; 1600 VEG, 2400 VEG, 3200 VEG [20]. We called this part of the analysis the “*whole diet*” study, given that it analyzed the whole composition of the diet.

#### 2.2.2. “*Delta*” Study

In order to better evaluate the differences among the corresponding impacts of the three patterns, we focused only on the difference in food components, limiting the analysis to the 2400 kcal patterns. We called this part of the analysis the “*delta*” study, given that it was based only on the delta, the difference, among the three patterns: it allowed us to focus on the consequences on the overall environmental impact of apparently small modifications in the composition of a diet.

As mentioned above, USDA DG propose eating patterns which, even in the OMN and LOV patterns, are largely based on plant foods. Therefore, each of the three patterns has a major overlapping area of plant food content (about 81% in mass). As a consequence, the differences in the environmental impacts of the three patterns stem only from the 19% in mass in which the three recommended eating patterns differ, mainly in the different protein sources. To obtain the food component of the *delta* study, for each of the three patterns the overlapping component have been subtracted, *i.e.*:

1. the “fruits”, “vegetables” and “grains” groups;
2. the total amounts of nuts, seeds, soy products listed in the OMN patterns;
3. the total amount of oils listed in the VEG patterns.

Therefore, for OMN patterns, the delta component included seafood, meat, poultry, eggs, dairy, oils; for LOV patterns, the delta component included eggs, beans and peas (in addition to the amount recommended in the “vegetable” group), soy products, nuts and seeds, dairy, oils; for VEG patterns, the delta component included beans and peas (in addition to the amount recommended in the “vegetable” group), soy products, nuts and seeds, non-dairy substitutes.

### 2.3. Sensitivity

In a previous paper we performed an accurate sensitivity analysis, showing that, for this kind of evaluation, any variation of the examined data elicited a variation in the results that came to be perceptually acceptable and unable to modify the interpretation of the results [16].

More recent studies from other authors confirmed the significant stability of LCA methodology when applied to the analysis of dietary patterns [41].

## 3. Results and Discussion

### 3.1. “Whole Diet” Study

The results obtained for the three food patterns (whole diet study), according to each method of analysis (indicator), are reported in Table 1.

The analysis results are expressed in points for week (*Pt*/*week*): the higher the “score” in *Pt*, the higher the damage to the environment.

**Table 1:** Whole diet study: total and partial environmental impacts (indicated as single score, *Pt* per week) of the three complete food patterns, according to each indicator.

Pattern/kcal	VEG 1600	VEG 2400	VEG 3200	LOV 1600	LOV 2400	LOV 3200	OMN 1600	OMN 2400	OMN 3200
*Ecoindicator99*									
Total	0.64800481	0.94997163	1.3071604	2.3859409	2.6923735	3.0492296	3.7122608	4.40574	4.9341334
Carcinogens	0.00019843	0.000286384	0.00036383	0.00133281	0.00142583	0.00150253	0.001630978	0.00180755	0.001925124
Respiratory organics	0.00018305	0.000251572	0.0003258	0.00030875	0.00037811	0.00045206	0.000417358	0.000518085	0.000606523
Respiratory inorganics	0.10049695	0.14412463	0.18855662	0.17767523	0.22237083	0.26670832	0.28722378	0.36469605	0.42409415
Climate change	0.03919139	0.055214859	0.0709742	0.13229725	0.14852923	0.16416739	0.17863998	0.20869219	0.23059325
Radiation	0.00002247	3.42 × 10^−5^	4.34 × 10^−5^	0.00038047	0.00039248	0.00040169	0.000398937	0.000416055	0.000427788
Ozone layer	0.00001066	1.47 × 10^−5^	1.90 × 10^−5^	3.40 × 10^−5^	3.85 × 10^−5^	4.27 × 10^−5^	5.57 × 10^−5^	6.64 × 10^−5^	7.36 × 10^−5^
Ecotoxicity	0.00106054	0.001434384	0.00171095	0.0026162	0.00302211	0.00329787	0.003002629	0.003519975	0.003860926
Acidification/Eutrophication	0.02526500	0.036874031	0.04925412	0.04261296	0.05475953	0.0671279	0.090794324	0.11735622	0.13629427
Land and water use	0.29042511	0.43349405	0.63636379	1.0764882	1.2228196	1.4256888	2.117066	2.5657137	2.9009677
Minerals	0.00006435	9.65× 10^−5^	0.00012433	0.00053008	0.00056272	0.00059049	0.000579085	0.00062538	0.000659985
Fossil fuels	0.19108686	0.27814636	0.35942432	0.95166495	1.0380746	1.1192498	1.0324519	1.1423284	1.2346302
*Ecopoint*									
Total	6962.153	9977.3835	13418.176	13599.654	16700.341	20134.632	26697.481	33726.743	38929.907
NOx	1190.3383	1687.0413	2174.7931	2741.075	3239.3288	3725.3904	3351.1462	4029.0262	4599.7719
SOx	769.90348	1087.0439	1383.1478	1016.3942	1333.0982	1628.8733	1078.1917	1416.3569	1723.7377
NMVOC	139.32288	192.98861	253.43336	273.88218	328.37026	388.55252	350.76288	427.2963	497.94655
NH3	842.99251	1250.5947	1703.2695	1192.126	1627.3129	2079.9873	3490.5891	4614.2057	5379.5064
Dust PM10	9.0847823	12.923818	15.191499	65.534052	69.802741	72.053644	72.707679	79.03103	82.418179
CO2	1377.9895	1943.5007	2498.664	4725.9375	5298.3987	5849.4224	6340.5217	7394.1754	8163.2418
Ozone layer	0.6610465	0.90966159	1.1767778	2.1511266	2.4272521	2.689679	3.4930708	4.1519191	4.5977102
Pb (air)	0.32147787	0.47727671	0.60871325	1.5374657	1.702998	1.833917	2.0107655	2.3081678	2.5067236
Cd (air)	1.5009171	2.1788895	2.7819723	9.748633	10.451046	11.049965	11.405062	12.571727	13.398671
Zn (air)	0.34491482	0.51571004	0.66441941	1.0479235	1.2308866	1.3790125	1.6420776	1.9914576	2.2250812
Hg (air)	1.1709015	1.7566871	2.2428252	11.655909	12.243954	12.729547	12.321511	13.092723	13.669511
COD	4.5777467	6.6868027	8.6133306	25.25851	27.433316	29.349171	30.326905	33.929372	36.545081
P	466.47307	688.61262	932.40825	605.62586	831.28441	1075.0789	1216.5565	1613.6763	1938.9971
N	2080.5034	2992.2043	4299.9698	2621.3589	3578.1404	4885.9003	10,403.361	13,712.094	16,063.676
Cr (water)	0.18439044	0.27280425	0.34372097	1.9087695	2.0000171	2.0706068	2.1334244	2.2856304	2.3864404
Zn (water)	0.11026105	0.16221025	0.20594354	0.94313822	0.99673879	1.0402484	1.0679982	1.1560351	1.2165352
Cu (water)	0.15052732	0.22462302	0.28297967	1.7211955	1.7973628	1.855504	1.9056096	2.0314536	2.1143221
Cd (water)	0.18614116	0.27169122	0.35127599	0.55325881	0.64589623	0.72481966	0.91269133	1.1064944	1.2360639
Hg (water)	0.7057345	1.0552604	1.3499184	5.9398762	6.286672	6.5811908	6.2440261	6.6747297	7.0105413
Pb (water)	0.0305093	0.045206483	0.05642818	0.30565965	0.32090765	0.33209206	0.33619951	0.35977884	0.37519415
Ni (water)	0.02068235	0.030679134	0.03847235	0.23622184	0.24660089	0.25435586	0.26447911	0.28248352	0.29403315
AOX (water)	0.00530489	0.007279036	0.00937398	0.01604704	0.01823847	0.02029386	0.027071622	0.032408888	0.03596629
Metals (soil)	0.21573554	0.31068211	0.39921194	1.1959458	1.2942133	1.382073	1.433325	1.5988108	1.7190422
Pesticide (soil)	15.523208	20.69761	25.872013	15.523208	20.69761	25.872013	15.523208	20.69761	25.872013
Energy	59.835651	86.870388	112.30158	277.97726	304.81086	330.2091	302.59665	336.61044	365.40786
*EDIP*									
Total	0.013394846	0.018757951	0.02442873	0.03742483	0.0429144	0.04856047	0.049088143	0.057968728	0.065202436
Global warming (GWP 100)	0.001047775	0.001477754	0.00189988	0.00359432	0.0040296	0.00444857	0.004823125	0.005624618	0.006209534
Acidification	0.000544453	0.000788231	0.00104088	0.00087095	0.00112338	0.00137577	0.001664129	0.002154371	0.002515469
Eutrophication	0.003267925	0.004453371	0.00574918	0.00361988	0.00482479	0.00611978	0.006304814	0.008318734	0.00997571
Photochemical smog	0.000170838	0.000235238	0.0003047	0.0003023	0.0003675	0.0004367	0.000415924	0.00051399	0.000598279
Ecotoxicity water chronic	0.001493251	0.002078688	0.00284514	0.00456145	0.00519663	0.00595413	0.007208578	0.008596149	0.009714592
Ecotoxicity water acute	0.001243991	0.001714344	0.0022113	0.00420647	0.0047279	0.00521615	0.006717897	0.00795496	0.008786304
Ecotoxicity soil chronic	0.002577418	0.003584406	0.00468446	0.00595102	0.00695597	0.0080556	0.006466586	0.00761594	0.00878632
Human toxicity air	0.000509268	0.000720343	0.00092095	0.00152606	0.00173505	0.00193547	0.001620564	0.001857016	0.002070554
Human toxicity water	7.44 × 10^−5^	0.000110764	0.00014493	0.00061465	0.00065113	0.00068526	0.000657524	0.000705894	0.00074591
Human toxicity soil	0.002465551	0.003594811	0.00462731	0.01217773	0.01330246	0.01433303	0.013209003	0.014627056	0.015799764

For all indicators, the results indicated that VEG patterns always had the lowest single score: LOV patterns had single scores of 3 ± 0.7 times higher than VEG patterns, and OMN patterns had single scores of 4.7 ± 1 times higher than VEG patterns, depending to the calorie intake.

### 3.2. “Delta” Study

The results obtained in the delta study, according to each of the above-mentioned indicators, are reported in Table 2.

**Table 2:** Delta study: total and partial environmental impacts (indicated as single score, *Pt* per week) of the 19% component in which the three 2400 kcal patterns differ (delta), according to each indicator.

Pattern	VEG Delta	LOV Delta	OMN Delta
*Ecoindicator99*			
Total	0.21163256	1.9540345	3.6643576
Carcinogens	8.05 × 10^−5^	0.001219979	0.001596435
Respiratory Organics	4.01 × 10^−5^	0.000166618	0.000305573
Respiratory Inorganics	0.021479271	0.09972547	0.24028832
Climate change	0.011307043	0.10462142	0.16357382
Radiation	4.92 × 10^−5^	0.000363216	0.000386427
Ozone layer	7.65 × 10^−5^	3.15 × 10^−5^	5.91 × 10^−5^
Ecotoxicity	0.00015814	0.001745867	0.002227464
Acidification/Eutrophication	0.005841891	0.023727391	0.08568815
Land and water use	0.14422255	0.93354814	2.2793129
Minerals	1.37 × 10^−5^	0.000479934	0.000541406
Fossil fuels	0.028476768	0.78840499	0.89037801
*Ecopoint*			
Total	1966.647	8736.5711	25,386.999
NOx	244.3271	1792.9067	2564.5648
SOx	100.58787	345.87779	424.46498
NMVOC	40.724424	175.43345	272.92316
NH3	210.51048	584.99251	3527.0838
Dust PM10	2.9942366	59.304326	68.263183
CO2	398.16059	3813.7786	5905.1117
Ozone layer	0.46604541	1.9578271	3.640162
Pb (air)	0.08821719	1.3213139	1.9105317
Cd (air)	0.53593394	8.5764778	10.615507
Zn (air)	0.092202355	0.8064707	1.5367188
Hg (air)	0.17861332	10.371796	11.187239
COD	1.3626347	22.224598	28.637096
P	107.57917	249.26705	1025.8589
N	849.72417	1431.9097	11270.471
Cr (water)	0.045675248	1.7736143	2.0564881
Zn (water)	0.029915182	0.85359468	1.0089572
Cu (water)	0.032329539	1.5634239	1.7889283
Cd (water)	0.07970724	0.46663514	0.92955853
Hg (water)	0.081921167	5.3177828	5.7018097
Pb (water)	0.006726089	0.28355506	0.32204421
Ni (water)	0.00528008	0.22105271	0.25655257
AOX (water)	0.003837047	0.014917741	0.029135601
Metals (soil)	0.078326533	1.0618578	1.3636445
Energy	8.9515701	226.28606	257.2731
*EDIP*			
Total	0.003713746	0.027870192	0.042734028
Global warming (GWP 100)	0.000297634	0.002849477	0.004412835
Acidification	0.000112545	0.000447691	0.001467579
Eutrophication	0.000333923	0.000705344	0.004129094
Photochemical smog	3.79 × 10^−5^	0.000170155	0.000315601
Ecotoxicity water chronic	0.001190023	0.004307961	0.00768123
Ecotoxicity water acute	0.000872404	0.003885964	0.007087624
Ecotoxicity soil chronic	0.000396519	0.003768079	0.004424933
Human toxicity air	4.81 × 10^−5^	0.001062836	0.00118261
Human toxicity water	1.70 × 10^−5^	0.000557353	0.000611296
Human toxicity soil	0.000407688	0.010115332	0.011421227

For all indicators, the results of the delta study showed that, compared with the single score of the VEG pattern, the single score of the LOV pattern was up to 9.2 times higher, and the single score of the OMN pattern was up to 17.3 times higher. The single score of the delta study for the OMN pattern (2400 kcal), for each indicator, was: 3.66 (Ecoindicator99), 25,387 (Ecopoint) and 0.043 (EDIP). It was higher than (or equal to, for LOV-EDIP) the single score calculated in the whole diet study for both the other diets, that was, respectively: 0.95 (VEG) and 2.69 (LOV), calculated with the Ecoindicator99; 9,977 (VEG) and 16,700 (LOV), calculated with the Ecopoint; 0.019 (VEG) and 0.043 (LOV), calculated with the EDIP.

### 3.3. “Whole Diet” Study *vs.* “Delta” Study

It can be noticed that the animal food component in the OMN pattern, while making up only 19% of the total weight of the diet, accounted for about 73%–83% of its total environmental impact: for Ecoindicator99 the single score was 3.66 (delta) out of 4.41 (whole); for Ecopoint, 25,387 (delta) out of 33,726 (whole); for EDIP, 0.043 (delta) out of 0.058 (whole).

Other interesting results were the ratios of the single score of the delta study to the single score of the whole diet study: 0.83 (OMN), 0.73 (LOV), and 0.22 (VEG). Moreover, the ratios between the single scores of the delta study over the single score of the plant food common component of the diet (81% in mass) was shown to be 1.21 (OMN), 0.98 (LOV), and 0.30 (VEG).

### 3.4. Distribution of the Sources of Relative Impact within the Dietary Patterns

The different components of the overall environmental impacts, accordingly to each indicator, within the same dietary pattern, can be summarized as follows:

#### 3.4.1. Ecoindicator99

The results obtained using Ecoindicator99 showed that the major impact, from 45% to 60% of the overall impact, always stemmed from land and water use. The second largest impact, from 25% to 50% of the total, came from energy use. The third cause of impact, from 7% to 16% of the total, was due to the emission of toxic inorganic compounds into the environment. Effects on climate change (5%–6%) and on acidification/eutrophication (2%–4%) represented another substantial impact.

#### 3.4.2. Ecopoint

The results of the Ecopoint analysis showed that the most impacting factor, from 35% to 55% of the total, was the contamination from inorganic nitrogen and phosphorous compounds. The second major cause of impact, from 21% to 37% of the total, was the emissions into the atmosphere, while the third source of impact, from 16% to 27% of the total, was due to the emission of oxides into the atmosphere. The inclusion of N-oxides among the Greenhouse Gas (GHG) subcategories put the GHG emissions at the same level of impact of inorganic nitrogen and phosphorous compounds (34%–57%).

#### 3.4.3. EDIP

The impacts detected by the above two indicators translated into a considerable number and variety of toxic impacts, that could be further evaluated by the EDIP indicator. Results of EDIP analysis showed that the highest impact, from 25% to 33% of the total, was due to human toxicity caused by soil contamination. The second-highest impact, from 20% to 29% of the total, was due to acute and chronic eco-toxicity of the water. The third-highest impact, from 13% to 19%, was due to chronic eco-toxicity of the soil. The fourth-highest impact, from 10% to 18%, was due to water eutrophication, while the fifth impact, from 8% to 10%, was given by various contributions to global warming.

### 3.5. Absolute Values of the Impacts in the Different Dietary Patterns

The above mentioned percentages represented the proportions of the various kinds of impact within the same dietary pattern, and they were very similar for all the examined dietary patterns, that is, for each dietary pattern its main impact was land and water use, then energy and so on.

But when taking into consideration the absolute values of those impacts, they varied dramatically among the various patterns. In fact, the total impact (single score) for the VEG pattern was 35% and 22%, respectively, than the one for the LOV and OMN patterns (these data apply specifically to the 2400 kcal scenario for Ecoindicator99). Therefore, even if, for example, the land and water use accounted for 50% of the impact both in VEG and in OMN diets, the absolute value in the VEG diet was 78% lower than the absolute value for the OMN diet for the same impact.

The overall results of our study showed that OMN diets had the highest impact, while VEG diets had the lowest environmental impact, independently of the calorie intake.

In most cases, the differences were just as significant also between a LOV and an OMN diet, so much so that the overall impact of a 3200 kcal LOV was always lower than that a 1600 kcal OMN one. The presence of animal food in the diet resulted to be the main impacting factor.

### 3.6. Subcategories of Impact

The study of the total impacts (represented by the single score, *Pt/week*) included not only the analysis of some well-known, commonly used impact subcategories, *i.e*., GHG, land and water use, but also other less commonly studied impact subcategories, all listed in Table 1 and Table 2: the various subscores of impact subcategories contributed to the final value of the single score of the respective indicator. Although more complex, the single score represents an index of the total environmental impact of food production, more accurate and comprehensive than the score of each single impact subcategory. For example, for the 2400 kcal OMN diet, the subscore for land and water use, a subcategory of Ecoindicator99, was respectively 5.92 and 2.10 times the score of the VEG and LOV patterns for the whole study (Table 3), and 15.80 and 2.44 for the delta study (Table 4). For comparison, the single score of the total impact category analyzed by Ecoindicator99 was 4.64 and 1.64 times for the whole study and 17.31 and 1.88 times for the delta study. It is worth to underline that in the whole diet study, land and water use subscore contributed for 45%–46% in the vegetarian patterns and 58% in the OMN patterns, to the total impact, as represented by the value of the single score (Table 3). Similarly, a recent study conducted in Germany by Meier *et al.* [42], which analyzed consumption data derived from a National Nutrition Survey, showed a land-saving potential effect of plant-based diets, which was maximum for VEG diets.

**Table 3:** Whole diet study: land and water use, and GHG subscores (indicated as single score, *Pt* per week) of the three complete food patterns, according to each indicator.

Pattern	VEG			LOV			OM		
kcal	1600	2400	3200	1600	2400	3200	1600	2400	3200
*Ecoindicator99*									
TOTAL single score	0.64800	0.94997	1.30716	2.38594	2.69237	3.04923	3.71226	4.40574	4.93413
*versus* VEG	-	-	-	-	-	-	573%	464%	377%
*versus* LOV	-	-	-	-	-	-	156%	164%	162%
*versus* OMN	17%	22%	26%	64%	61%	62%	-	-	-
Land and water use subscore	0.29043	0.43349	0.63636	1.07649	1.22282	1.42569	2.11707	2.56571	2.90097
*versus* TOTAL	45%	46%	49%	45%	45%	47%	57%	58%	59%
*versus* VEG	-	-	-	-	-	-	729%	592%	456%
*versus* LOV	-	-	-	-	-	-	197%	210%	203%
*versus* OMN	14%	17%	22%	51%	48%	49%	-	-	-
Climate change subscore	0.03919	0.05521	0.07097	0.13230	0.14853	0.16417	0.17864	0.20869	0.23059
*versus* TOTAL	6%	6%	5%	6%	6%	5%	5%	5%	5%
*versus* VEG	-	-	-	-	-	-	456%	378%	325%
*versus* LOV	-	-	-	-	-	-	135%	141%	140%
*versus* OMN	22%	26%	31%	74%	71%	71%	-	-	-
*Ecopoint*									
TOTAL single score	6962.1	9977.3	13,418.1	13,599.6	16,700.3	20,134.6	26,697.4	33,726.7	38,929.9
*versus* VEG	-	-	-	-	-	-	383%	338%	290%
*versus* LOV	-	-	-	-	-	-	196%	202%	193%
*versus* OMN	26%	30%	34%	51%	50%	52%	-	-	-
Greenhouse Gas (GHG) subscore	2707.6	3823.5	4926.8	7740.8	8866.0	9963.3	10,042.4	11,850.4	13,260.9
*versus* TOTAL	39%	38%	37%	57%	53%	49%	38%	35%	34%
*versus* VEG	-	-	-	-	-	-	371%	310%	269%
*versus* LOV	-	-	-	-	-	-	130%	134%	133%
*versus* OMN	27%	32%	37%	77%	75%	75%	-	-	-
*EDIP*									
TOTAL single score	0.01339	0.01876	0.02443	0.03742	0.04291	0.04856	0.04909	0.05797	0.06520
*versus* VEG	-	-	-	-	-	-	366%	309%	267%
*versus* LOV	-	-	-	-	-	-	131%	135%	134%
*versus* OMN	27%	32%	37%	76%	74%	74%	-	-	-
Global warming (GWP 100) subscore	0.00105	0.00148	0.00190	0.00359	0.00403	0.00445	0.00482	0.00562	0.00621
*versus* TOTAL	8%	8%	8%	10%	9%	9%	10%	10%	10%
*versus* VEG	-	-	-	-	-	-	460%	381%	327%
*versus* LOV	-	-	-	-	-	-	134%	140%	140%
*versus* OMN	22%	26%	31%	75%	72%	72%	-	-	-

**Table 4:** Delta study: land & water use and GHG subscores (indicated as single score, *Pt* per week) of the 19% component in which the three 2400 kcal patterns differ (delta), according to each indicator.

Pattern	VEG	LOV	OMN
*Ecoindicator99*			
TOTAL single score	0.21163	1.95403	3.66436
*versus* VEG	-	-	1731%
*versus* LOV	-	-	188%
*versus* OMN	6%	53%	-
Land and water use subscore	0.14422	0.93355	2.27931
*versus* TOTAL	68%	48%	62%
*versus* VEG	-	-	1580%
*versus* LOV	-	-	244%
*versus* OMN	6%	41%	-
Climate change subscore	0.01131	0.10462	0.16357
*versus* TOTAL	5%	5%	4%
*versus* VEG	-	-	1447%
*versus* LOV	-	-	156%
*versus* OMN	7%	64%	-
*Ecopoint*			
TOTAL single score	1966.6	8736.5	25,386.9
*versus* VEG	-	-	1291%
*versus* LOV	-	-	291%
*versus* OMN	8%	34%	-
Greenhouse Gas (GHG) subscore	683.2	5782.1	8742.5
*versus* TOTAL	35%	66%	34%
*versus* VEG	-	-	1280%
*versus* LOV	-	-	151%
*versus* OMN	8%	66%	-
*EDIP*			
TOTAL single score	0.00371	0.02787	0.04273
*versus* VEG	-	-	1151%
*versus* LOV	-	-	153%
*versus* OMN	9%	65%	-
Global warming (GWP 100) subscore	0.00030	0.00285	0.00441
*versus* TOTAL	8%	10%	10%
*versus* VEG	-	-	1483%
*versus* LOV	-	-	155%
*versus* OMN	7%	65%	-

GHG emissions (calculated as GHG emissions in kilograms of carbon dioxide equivalents, kgCO_2_e), have been assessed in two recent studies in UK and Northern USA [43,44]. In the UK study, the real 2000 kcal diet of 55,504 subjects was analyzed [43], and the average production of kgCO_2_e/day resulted to be, in medium-meat-eaters, 1.95 and 1.48 times the amounts produced by VEG and LOV subjects, respectively. Soret [44] reported similar results for the average emissions of CO_2_e per year in 73,308 American nonvegetarians, respectively 1.41 and 1.28 times the amounts produced by vegetarians and semivegetarians, for an average calorie intake of about 1700 kcal.

In our LCA study, GHG emissions were analyzed by the indicators Ecoindicator99 (climate change subcategory), Ecopoint (NO*x*, NMVOC-Non Methane Volatile Organic Compounds, CO_2_ subcategories) and EDIP (global warming subcategory), and contributed to the single score of the respective indicator for 5%–6% (Ecoindicator99), 34%–57% (Ecopoint) and 8%–10% (EDIP). For the lower calorie patterns, the 1600 kcal diets, the subscore for GHG emissions in OMN pattern was respectively: 4.56 and 1.35 times the score of the VEG and LOV patterns in Ecoindicator99; 3.71 and 1.30 times the score of the VEG and LOV patterns in Ecopoint; 4.60 and 1.34 times the score of the VEG and LOV patterns in EDIP. Again, it is worthwhile to underline that in the 1600 kcal OMN pattern, the single score of the total impact categories was 5.73 and 1.56 times (Ecoindicator99), 3.83 and 1.96 times (Ecopoint) and 3.66 and 1.31 times (EDIP) the single score of the VEG and LOV patterns, respectively. The data referred to the above mentioned subcategories, for all the dietary patterns and all the indicators, were summarized in Table 3 and Table 4.

Although the importance of the above mentioned studies relies on the analysis of real diets, they have been performed with an approach different from the present study. In fact, our study evaluated theoretical diets, so we did not consider any difference in geographical zone or transportation, import-export food fluxes and related emissions during cooking and storing in the household/in restaurants. For these reasons, we think that a comparison among the results of the different studies is not possible, even if these studies on real diets confirm the lowest environmental impact of plant based diets: future studies, performed in other countries and evaluating the total impacts, are warranted.

## 4. Conclusions

The results of our study confirmed the findings reached over the last few years by the research in this field, and showed that the environmental impact of a diet is mainly related to the consumption of animal products. This is the main reason why the total environmental impact of various dietary patterns can differ so much, as we found in our study. This is true from every perspective: climate change, energy consumption, water requirements, waste disposal, soil usage, deforestation, chemical use, and impacts both environmental and social aspects—namely the possibility of feeding all the world’s citizens.

The United Nations’ UNEP report, “Assessing the Environmental Impacts of Consumption and Production”, urges a global move towards an animal-product-free diet, identifying animal product consumption as one of the primary sources of environmental impact, pollution, greenhouse effect and resource waste. Factory farming is among the first four sectors labeled in the report as “First Priority”, and we find meat and dairy processing in the first positions of the “Second Priority” sectors. The report conclusions recommend a “substantial worldwide diet change, away from animal products” in order to reach a sustainable food production and to be able to feed an increasing human population [45].

From the viewpoint of human health, the USDA DG implicitly lead in the same direction: the environmental impacts of the various diets proposed by the DG are relatively low, exactly because they contain a high proportion of plant food and a very limited amount of animal food; basically, they recommend a dietary pattern much more slanted towards a direct consumption of plant foods than the average dietary pattern followed by people in industrialized countries all over the world, whose animal food consumption is much higher than the USDA DG recommends [16,20,28].

In relation to this, it is important to notice that the dietary OMN and LOV USDA patterns are quite different from the most widespread and common dietary patterns followed by people in industrialized countries all over the world: the average person’s consumption of animal food is much higher than that recommended by the DG [16]. As a consequence, there is likely to be a considerable difference between the estimated environmental impact of the “ideal” OMN and LOV diets recommended by the USDA DG and the impact of the “real” diets followed by most people in industrialized countries. This difference should be carefully assessed in future studies performed in different countries, as recently done in Germany [42], UK [43], and USA [44].

The composition of the healthy OMN and LOV diets recommended by the USDA DG is evidence of the desirability of a shift towards a much higher consumption of plant food, and a correspondingly much lower consumption of animal food, not only to reduce environmental impacts but also for health reasons. The consequences of a radical shift towards a plant-based diet would be many, all of them positive: a substantial influence on climate change, a profitable decrease in energy use and water waste, a lessening of the impact of deforestation, a much more rational use of soil (also leading to a dramatic decrease of chemicals use in agriculture).

The 2010 USDA DG should stimulate not only the scientific community but also national governments, international and scientific institutions, and the media, to promote a cultural shift: there is much that national and worldwide institutions, and the scientific community itself, can do in order to speed up the transition towards more environmentally sustainable, and healthier, dietary habits.
